# Creatine kinase levels, carcass, and physicochemical properties of breast meat from traditionally slaughtered village chickens

**DOI:** 10.1007/s11250-025-04390-y

**Published:** 2025-03-19

**Authors:** Busisiwe Gunya, Zwelethu Mfanafuthi Mdletshe

**Affiliations:** Department of Agricultural Economics and Animal Production, Faculty of Science and Agriculture, University of Limpopo, Private Bag X1106, Sovenga, 0727 South Africa

**Keywords:** Transverse neck incision, Village chickens, Creatine kinase levels, Physicochemical properties

## Abstract

The objective of the study was to determine the effect of sex, season, and breed on creatine kinase (CK) levels, carcass, and the physicochemical properties of village chickens slaughtered using the transverse neck incision (TNI). One hundred chickens were stratified based on sex, breed, and season and were randomly assigned to different treatment groups to assess their live weight, carcass weight, meat quality, and CK levels. Results indicated that sex influenced live and carcass weights, with cocks (*P < *0.05) being heavier than hens. Seasonal variations had a more significant effect on weights and meat quality, with the hot-dry season yielding the highest (*P < *0.05) weights and the rainy season the lowest (*P < *0.05). Seasonal changes also affected (*P < *0.05) pH, meat colour, cooking loss, and drip loss, creatine kinase levels. The Leghorn chickens exhibited the highest (*P < *0.05) live weight, and the Australorp had the lowest (*P < *0.05). The Orpington breed produced the firmest meat (*P < *0.05), whereas the Australorp yielded the tenderest. The study suggests that season and genetic factors significantly influence meat quality in village chickens. Further research is recommended into the nutritional status and muscle fibre composition to enhance meat quality. These findings offer valuable insights for improving slaughter practices and meat production in resource-limited farming systems.

## Introduction

In certain regions and cultures, traditional slaughter methods without stunning, such as halal or kosher practices, remain prevalent, particularly for religious and cultural reasons. This persists despite stunning being the standard in many areas of the world to reduce animal suffering (Fayemi and Muchenje 2012; Mdletshe et al. 2021; Gregory 2005). Some consumers prefer meat from animals slaughtered in these traditional ways, as they believe it aligns with their ethical, cultural, or religious beliefs and results in high-quality meat. Village chickens, common in resource-limited households and significant for income and nutrition, are often slaughtered using these traditional methods (Mwale and Masika, 2009). However, the productivity of village chickens is constrained by inadequate management practices and stressful situations such as informal slaughter, which can negatively affect their meat quality.

Informal slaughter of village chickens without stunning is one of the limiting factors to physical stress and meat quality of village chicken breast meat (Fayemi and Muchenje 2012). The metabolic pathway varies with genotype, sex, and season (Wattanachant 2008). In Southern Africa, the transverse neck incision (TNI) is the common informal slaughter method for chickens. The slaughter method is permitted by the South African Meat Safety Act of 2004, No 40, subsection 7 (a) and (b). The slaughter method has been associated with high antemortem stress, causing a significant depletion of muscle glycogen reserves, reducing substrate availability for anaerobic glycolysis at post-mortem, the extent to which myoglobin is oxygenated in the muscle, therefore affecting meat pH, colour (L*, a*, and b*), water holding capacity (WHC), cooking loss (CL), and drip loss (DL) (Wang et al. 2022, 2023). Determining the likely differences in creatine kinase levels and physicochemical properties of breast meat due to genotype, sex, and season could assist in the formulation of sustainable interventions for improving slaughter practices, and the physicochemical properties of breast meat for the South African Indigenous chicken breeds.

Elevated creatine kinase levels in the bloodstream are associated with high physical stress during the slaughter process, muscle breakdown, and subsequent muscle glycogen depletion, which is converted to lactate hydrogen, releasing ions responsible for muscle acidification post-mortem. This results in dark, firm, and dry (DFD) meat, which has a higher water-holding capacity, lower cooking and drip loss, and reduced tenderness (Miller et al. 2001; Wattanachant 2008; Ngambu 2011). The creatine kinase (CK) levels in the bloodstream indicate physical stress during the slaughter process, reflecting how the animal’s body responds to it, alongside muscle breakdown and physicochemical properties (Chulayo and Muchenje 2013). However, limited information exists on the interaction between genotype, sex, and season regarding the slaughter of Indigenous chicken breeds using TNI, particularly about creatine kinase levels in the blood and the physicochemical properties of breast meat.

Understanding the effect of TNI slaughter methods on creatine kinase levels can empower resource-limited households engaged in communal farming to develop effective genotype, sex, and seasonal slaughter practices that enhance the physicochemical properties of breast meat and promote welfare standards for South African Indigenous chicken breeds. The objective of the article is to determine how sex, genotype, and season influence creatine kinase levels and the physicochemical properties of breast meat in village chickens slaughtered using the transverse neck incision (TNI). The null hypothesis being tested posits that there will be no significant differences in the creatine kinase levels and physicochemical properties among different genotypes, sexes, and seasons.

## Materials and methods

### Birds, experimental design and treatment

A total of one hundred village chickens, comprising different sexes (48 cocks and 52 hens) and breeds, were purchased from local households and randomly assigned to a treatment group of sex and breed for physiological and physiochemical evaluation across seasons. The breeds purchased included Austrolorp (*n =* 5), Cornish (*n =* 11), Hampshire (*n =* 25), Leghorn (*n =* 13), Barred Plymouth (*n =* 12), Orpington (*n =* 30), and Turken (*n =* 4). Chickens were collected a day before slaughter and deprived of food while being given water ad libitum for 12 h to facilitate handling and recovery from the stress of transportation. They were tagged with coloured leg bands, stratified based on sex and breed, and randomly assigned to seasons. Slaughtering and sampling were done in June for the cool-dry season, September for the hot-dry season, December for the rainy season, and March for the Post-rainy season. Chickens were weighed before slaughter using a digital scale (Manual Poultry Scale BAT1, Kempton Park, South Africa) to determine their live weights. Criteria for identifying indigenous breeds of Southern Africa for the study followed by Hlokoe and Tyasi (2022).

### Compliance with ethical clearance

The Animal Ethics Committee of the University of Fort Hare (MUC01 ISGUN01) approved the experiment following the University guide, which is compliant with the international and South African National Standards (SANS 10386:2008) for the care and use of animals in research and teaching. A veterinarian registered with the South African Veterinary Council (SAVC) observed all slaughter procedures.

### Slaughtering of birds

Birds were slaughtered by two slaughtermen, permitted by the Meat Safety Act (2000) regulations of South Africa, where chickens were slaughtered without stunning for personal use, using the transverse neck incision, which requires the use of a sharp knife. All birds were slaughtered in the morning (0800 h). One slaughterman was responsible for holding legs and wings while the head was laid on the slaughtering slab. The second slaughterman held the head and severed the jugular veins, trachea and oesophagus using a sharp knife. Each cut was a change in the direction of movement of the knife. The neck was held horizontally to the slaughter slab until the bleeding was complete. After bleeding, the carcasses were scalded by submerging them in water between 70 $^\circ{\rm C}$ and 90 $^\circ{\rm C}$ for 30 s. Manual feather plucking was done. Following the evisceration of carcasses, a digital scale (Kern Weighing Scale, 6 kg Weight Capacity Type C—European Plug) was used to weigh the remains. Following the removal of breasts from the carcasses, they were deboned, vacuum-packed (Genesis 80GVS Vacuum Sealer), and stored in food-grade, high-density polyethene bags at 4 °C for 24 h to measure their physicochemical characteristics.

### Measurement of stress

#### Creatine kinase level determination

Blood was collected into the EDTA (Purple top EDTA Blood Collection Tube) tube while the chickens were bleeding. Blood samples were stored in ice until the plasma was separated within 2 h of collection. Blood samples were also centrifuged at 21 $^\circ{\rm C}$ for 20 min at 3550 rpm and placed in 1.5 ml tubes using the model 5403 centrifuge by Eppendorf GmbH for the complete separation of plasma. Creatine kinase level analysis was done using a model DXC 600. Creatine kinase Reagent for SYCHRON Systems (CK × 2) had reactive ingredients: Creatine Phosphate, Disodium salt, 461 mmol/L, Glucose 24.0 mmol/L, Glucose – 6-Phosphate Dehydrogenase 46.1 KU/L. All the ingredients were added to determine CK activity per litre (U/L) in plasma.

The offals were removed, and carcasses were weighed.

### Physicochemical properties

#### Determination of meat pH and colour

Meat pH was measured approximately 45 min, 24 and 48 h after slaughter using a portable pH meter (CRISON pH25, CRISON Instruments SA, Spain). Meat colour (L* = Lightness, a* = Redness and b* = Yellowness) was measured in triplicate at 45 min, 24 and 48 h after slaughter using a colour guide (45/0 BYK-Gardener GmbH machine) with a 20 mm diameter measurement area and illuminant D65-day light, 10º standard observer.

#### Determination of cooking loss and shear force

Freshly cut chicken breasts were weighed (initial weight) to form individual standardised slices 50 mm thick (maximum), and a standard weight in thin-walled plastic bags was placed in a warm water-bath, the bag opening extending above the water surface. Samples were cooked to a defined internal temperature of 85 °C for 45 min. When the end-point temperature had been attained, samples were removed from the water-bath and cooled. The meat was then taken from the bag, blotted dry and weighed. Cooking loss was calculated using the following formula:

$$Cooking\;loss = [(weight\;before\;cooked-- weight\;after\;cooked)\;\div\;weight\;before\;cooked] \times\;100$$

Following cooking, sub-samples of specified core diameter were parallel to the grain of the meat. Three sub-samples measuring 10 mm core diameter were cored parallel to the grain of the meat. The samples were sheared perpendicular to the fibre direction using a warner Bratzler Shear device mounted on Instron (Model (3344), Universal Testing apparatus. The mean maximum recorded for the three cores was presented as the average peak force in Newton’s (N) for each sample.

#### Determination of drip loss

Drip loss was assessed by following the method outlined by Honikel (1998) for evaluating the physical characteristics of meat. The breast samples of birds were placed on the tray for 24 h to remove fluid from the breast meat, and they were dried using a paper towel. The samples were dried twice. Drip loss was calculated as the weight of meat before drying minus the weight after drying. This was expressed as a percentage.

#### Statistical analysis

All data were analysed using statistical analysis software (SAS). A general linear model (GLM) was used to test the effect of sex, breed, and seasonal variation on creatine kinase levels, carcass (cold dressed weight, dressing percentage), and physicochemical properties (cooking loss, WB shear force, pH_u_, L*, a*, and b* values) of breast meat of village chickens that were slaughtered using the transverse neck incision. Mean separation was done using the LSD test option of SAS (2003).

The statistical model used was:

$$Yijkl=\mu +{A}_{i}+{G}_{j}+{S}_{1}+{{\left(A\times G\right)}_{ij}+\left(A\times S\right)}_{il}+{\left(G\times S\right)}_{jl}+{E}_{ikjl}$$

where Yijk = response variables (cold dressed weight, dressing percentage, pH, colour, tenderness, CL, CK, stress levels).

A_i_ = Sex.

G_j_ = Breed.

S_l_ = Season.

μ = population mean;

E_ikjl_ = residual error.

## Results

### Effect of sex on carcass characteristics

Sex-affected (*P < *0.05) carcass characteristics (Table 1). Cocks had higher live weight and carcass weight than hens. Sex did not significantly (*P >* 0.05) affect the dressing percentage of village chickens.

**Table 1:** Least square means (± standard error, SE) of carcass characteristics and the sex of village chickens for live weight, carcass weight, and dressing percentage

Carcass Characteristics	Cock	Hen
Live Weight (kg)	1.9 ± 0.07^b^	1.6 ± 0.05^a^
Carcass Weight (kg)	1.2 ± 0.05^b^	1.0 ± 0.03^a^
Dressing Percentage	64.9 ± 1.44	63.5 ± 1.11

^a^^,^^b^Means in the same row with different superscripts are significantly different (*P < 0.05)

### Effect of sex on creatine kinase, meat colour, pH, tenderness, cooking loss, drip loss

Sex did not affect (*P >* 0.05) L*, a* and b* of chickens (Table 2). Hens produced meat that had a higher L* value than cocks. Meat from cocks had higher values for a* than meat from hens. Meat from hens had higher values for b* than meat from cocks. Sex did not affect (*P >* 0.05) the CK levels, meat pH, WSBF values, cooking loss, and drip loss.

**Table 2:** Least mean squares (± SE) for meat attributes and sex of village chickens

Meat attributes	Cock	Hen
Cooking Loss (%)	19.1 ± 0.87	19.3 ± 0.66
Drip Loss (%)	14.2 ± 0.52	15.3 ± 0.40
Warner Bratzler Shear Force (N)	20.6 ± 2.22	20.5 ± 1.72
pH	6.2 ± 0.02	6.2 ± 0.01
Lightness (L*)	47.9 ± 0.73^a^	50.8 ± 0.54^b^
Redness (a*)	6.5 ± 0.24^a^	5.7 ± 0.18^b^
Yellowness (b*) Creatine Kinase (U/L)	14.9 ± 0.52^a^ 6768.0 ± 740.79	17.3 ± 0.39^b^ 7140.9 ± 610.29

^a^^,^^b^Means in the same row without the same superscripts are significantly different (*P < 0.05)

### The effect of season on carcass characteristics

Season-influenced (*P < *0.05) slaughter weight and carcass weight of chickens (Table 3). Chickens had the highest slaughter weight in the hot-dry season, while the lowest slaughter weight was observed in the rainy season. No seasonal differences were observed (*P < *0.05) in the dressing percentage.

**Table 3:** Least square means (± standard error) for village chicken carcass characteristics by season

Carcass Characteristics	Season			
	Hot-dry	Rainy	Post-rainy	Cool-dry
Live weight (kg)	2.1 ± 0.08^c^	1.5 ± 0.08^a^	1.8 ± 0.08^b^	1.7 ± 0.08^b^
Carcass weight (kg)	1.4 ± 0.06^c^	0.9 ± 0.06^a^	1.0 ± 0.06^ab^	1.1 ± 0.05^b^
Dressing %	65.6 ± 1.68	63.2 ± 1.65	62.3 ± 1.70	65.7 ± 1.69

^a^^,^^b^Means in the same row having different superscripts are significantly different (*P < 0.05)

### Effect of season on meat colour (L*, a* and b*), pH, tenderness, cooking loss, drip loss and creatine kinase

Season-influenced (*P < *0.05) meat pH, L*, a*, b*, WSBF, cooking loss, drip loss and CK level (Table 4). Meat from chickens slaughtered in the rainy season had the highest pH value, L* values, and drip loss values. The lowest pH values were found in chickens slaughtered in the cool-dry, while the lowest L* values were observed in chickens slaughtered in the hot-dry season. The highest a* values were observed in the hot-dry season and the lowest in the rainy season. The highest b* values and lowest b* values were observed in the cool-dry and the rainy seasons, respectively. The highest and lowest cooking loss values were observed in meat from chickens slaughtered in the hot-dry and cool-dry seasons, respectively. The lowest drip loss values were observed in breast meat from chickens slaughtered in the cool-dry season and the highest in the rainy season. Breast meat from chickens slaughtered in the cool-dry season had the highest WSBF values and CK levels, while the lowest WBSF values and CK levels were found in breast meat from chickens slaughtered in the hot-dry and cool-dry seasons.

**Table 4:** Least square means (± SE) for meat attributes and seasonal variations in village chickens

Meat attributes	Season			
	Hot-dry	Rainy	Post-rainy	Cool-dry
Cooking Loss (%)	21.7 ± 1.01^c^	19.0 ± 0.99^b^	19.5 ± 1.03^b^	16.8 ± 1.10^a^
Drip Loss (%)	11.9 ± 0.61^a^	18.8 ± 0.60^b^	17.7 ± 0.61^b^	10.4 ± 0.61^a^
WSBF (N)	16.0 ± 2.45^a^	19.8 ± 2.45^a^	16.7 ± 2.74^a^	29.8 ± 2.58^b^
pH	6.5 ± 0.03^c^	6.5 ± 0.03^c^	6.1 ± 0.03^b^	5.7 ± 0.03^a^
Lightness (L*)	44.0 ± 0.93^a^	55.9 ± 0.78^d^	46.9 ± 0.86^b^	50.5 ± 0.83^c^
Redness (a*)	7.0 ± 0.28^c^	3.5 ± 0.26^a^	6.2 ± 0.029^b^	7.6 ± 0.31^c^
Yellowness (b*)	16.6 ± 0.68^b^	13.6 ± 0.57^a^	15.2 ± 0.62^b^	18.9 ± 0.60^c^
Creatine Kinase (U/L)	4998.0 ± 770.80^a^	5586.0 ± 773.79^a^	7158.9 ± 775.03^a^	8119.9 ± 767.17^b^

^a^^,^^b^Means in the same row having different superscripts are significantly different (*P < 0.05)

### Effect of breeds on carcass characteristics

Breed affected (*P < *0.05) live weight and carcass weight of chickens (Table 5). The Autrolop, Cornish, and Hampshire breeds had the lowest live weight (*P < *0.05) compared to other breeds. The Plymouth breed had the highest carcass weight than other breeds, while Ausrolop had the lowest carcass weight. No breed differences were observed (*P >* 0.05) in the dressing percentage.

**Table 5:** Least square means (± standard error) for carcass characteristics of village chickens by breed

Breed	Live weight (kg)	Carcass weight (kg)	% Dressing
Austrolop (*n =* 5)	1.4 ± 0.17^a^	0.9 ± 0.11^a^	63.3 ± 3.40
Cornish (*n =* 11)	1.7 ± 0.11^ab^	1.1 ± 0.07^ab^	64.0 ± 2.27
Hampshire (*n =* 25)	1.7 ± 0.07^a^	1.1 ± 0.05^ab^	63.6 ± 1.53
Leghorn (*n =* 13)	2.0 ± 0.11^c^	1.2 ± 0.07^b^	61.1 ± 2.17
Plymouth (*n =* 12)	2.0 ± 0.11^c^	1.3 ± 0.08^c^	66.8 ± 2.27
Orpington (*n =* 30)	1.8 ± 0.07^bc^	1.2 ± 0.04^bc^	66.0 ± 1.43
Turken (*n =* 4)	1.8 ± 0.18^bc^	1.2 ± 0.13^bc^	64.7 ± 3.75

^a^^,^^b^Means in the same column having different superscripts are significantly different (*P < 0.05)

### Effect of breed on meat colour (L*, a* and b*), pH, tenderness, cooking loss, drip loss and creatine kinase

Table 6 shows the effects of chicken breeds on pH, L*, a*, b*, WBSF, cooking loss, drip loss, and CK levels. Breed had a significant effect (*P < *0.05) on L*, a*, b* WSBF of chicken. Orpington had the highest (*P < *0.05) WBSF values, while Austrolop had the lowest WBSF values. Breast meat from Hampshire had the highest (*P < *0.05) L* and b* values compared to other breeds. The Leghorn breed produced meat with the highest a* value of other breeds.

**Table 6:** Effect of chicken breed on physicochemical properties (colour, pH, tenderness, cooking, and drip loss) and creatine kinase

Breed	Cooking Loss (%)	Drip Loss (%)	WSBF(N)	pH	Lightness (L*)	Redness (a*)	Yellowness (b*)	Creatine kinase (L/U)
Australorp	19.6 ± 2.07	14.3 ± 1.24	13.4 ± 5.53^a^	6.2 ± 0.06	47.9 ± 1.67^ab^	5.7 ± 0.56^a^	16.1 ± 1.22^ab^	4885.0 ± 2333.56
Cornish	19.6 ± 1.37	14.4 ± 0.82	17.1 ± 3.42^b^	6.2 ± 0.03	50.3 ± 1.08^b^	5.6 ± 0.37^a^	16.9 ± 0.78^b^	7570.4 ± 1130.03
Hampshire	19.0 ± 0.93	15.2 ± 0.56	23.7 ± 2.17^c^	6.2 ± 0.03	53.9 ± 0.74^c^	5.7 ± 0.25^a^	17.6 ± 0.54^b^	6955.9 ± 753.21
Leghorn	18.6 ± 1.31	14.3 ± 0.78	19.9 ± 3.44^b^	6.3 ± 0.04	48.8 ± 1.14^ab^	6.7 ± 0.38^c^	16.4 ± 0.83^ab^	7926.2 ± 1240.16
Plymouth	18.5 ± 1.37	14.5 ± 0.82	23.5 ± 3.43^c^	6.2 ± 0.03	50.0 ± 1.07^b^	5.9 ± 0.36^a^	14.7 ± 0.78^a^	8325.4 ± 944.05
Orpington	19.4 ± 0.87	14.5 ± 0.52	24.2 ± 2.07^c^	6.2 ± 0.03	50.1 ± 0.70^b^	6.4 ± 0.23^bc^	16.8 ± 0.51^bc^	6455.9 ± 736.55
Turken	19.9 ± 2.26	15.7 ± 1.26	22.4 ± 1.99^c^	6.3 ± 0.06	44.4 ± 1.79^a^	6.4 ± 0.61^bc^	14.3 ± 1.31^a^	6565.7 ± 184.85

^a^^,^^b^Means in the same column with different superscripts are significantly different (*P < 0.05)

## Discussion

The objective of the article is to determine how sex, genotype, and season influence creatine kinase levels and the physicochemical properties of breast meat in village chickens slaughtered using the transverse neck incision (TNI). The observed heavier live weight for hens compared to cocks may be influenced by a higher growth rate, thus attaining a greater mature weight than females (Nthimo et al. 2004). These findings align with those reported by Thutwa et al. (2012) and Isidahomen et al. (2012). The observed lighter and yellower meat in cocks and darker breast meat in hens could be attributed to the fact that cocks possess type one muscle while hens have type two muscle; type one muscle is darker than type two. Consequently, cocks have darker meat, whereas hens present lighter meat. These findings are consistent with those of Salakova et al. (2009), who stated that meat from female chickens was pale and yellow compared to that from males.

The differences in live and carcass weights may stem from the various genotypes of breeds. These results correspond with the reports of Jaturasitha et al. (2008) and Olwamu et al. (2012), who indicated that the genotype of chickens plays a significant role in carcass characteristics. These findings corroborate previous studies (Martinez-Cerezo et al. 2001; Mancini and Hunt 2005), where the breed of chicken was shown to affect meat colour. The effect of breed on the lightness, redness, and yellowness of chickens may be due to differing genetic makeups (Wattanachant 2004). The higher live and carcass weights observed in hot-dry conditions and the lowest weights in the cool-dry season could be influenced by limited feed resources, leading to slow metabolism and reduced growth rates (Lwesya et al. 2004), resulting in denser muscles and firmer textures. These results align with the findings of Lwesya et al. (2004), who noted that village chickens’ live weights fluctuate with the seasons since they forage for feed. Further studies examining the nutritional status of chickens to determine live and carcass weights may be necessary.

The observed lower cooking loss may result from slower muscle growth, fat deposition, and tighter muscle fibres. Tighter muscle fibres contract less during cooking, thereby reducing the amount of water released. Furthermore, these observations could be explained by stress arising from feed shortages, leading to decreased muscle glycogen content. Low glycogen content results in a high ultimate pH, which increases space availability, allowing more water to be retained within myofibrillar proteins. In the cool-dry season, the observed higher water holding capacity (WSBF) may be attributed to slower muscle growth and less fat deposition, resulting in firmer muscle fibres due to denser fibres and a mature structure that is less well organised. These findings are akin to those of Bianchi et al. (2007), who stated that cooked breast shear force values were higher during the cool-dry season. A lack of feed availability could also explain such observations, as chickens tend to scavenge more for food during the cool-dry season. Wattanachant (2004) noted that the more village chickens scavenge, the more lactic acid builds up, resulting in a higher ultimate pH, which makes the meat less tender or tougher.

Meat pH and meat colour are positively correlated (Wattanachant 2008). Meat from village chickens exhibited the highest L* value in the rainy season and had the highest a* and b* values during the cool-dry season. These observations can be accounted for by high moisture content and slower growth and metabolic rate due to heat stress, resulting in breast meat with lower fat content. This causes the meat to appear lighter, reflecting light and creating a pale appearance (Berri 2000; Berri et al. 2005). During the cool-dry season, birds undergo dietary changes and exhibit reduced activity, leading to greater fat deposition for insulation. Dietary changes are also linked to the consumption of food rich in fats and carotenoids, resulting in meat with a yellow hue and a darker red appearance. Additionally, these observations may also be explained by a decrease in physical activity which leads to increased fat deposition for insulation and myoglobin content, contributing to the darker meat appearance and yellowness.

The observed elevated creatine kinase (CK) levels in the cool-dry season compared to the rainy seasons can be primarily attributed to increased muscle function as an effort to enhance energy production for insulation, as this enzyme aids in energy production while relying more on creatine phosphate as a quick energy source (Chulayo and Muchenje 2013; Chulayo and Muchenje 2013). Moreover, this could also be elucidated by the heightened activity of fast-twitch muscle fibres, which are energy-demanding (Weng et al. 2022). The observed lower shear force in breast meat from the Australorp compared to other breeds may stem from a higher concentration of fast-twitch muscle fibres and connective tissues, as they mature quickly, resulting in less tough meat (Weng et al. 2022). The observed lower lightness (a*) and yellowness (b*) in breast meat from Australorp, Leghorn, Plymouth, and Turken compared to other breeds could be explained by higher myoglobin content due to increased oxygen demand in the muscle arising from heightened muscle activity, which results in breast meat with a darker appearance.

## Conclusion

This study investigated the effect of sex, season, and breed on the creatine kinase levels, carcass, and physicochemical properties of village chickens. Sex influenced live and carcass weights, with cocks being heavier. Seasonal variations had a more pronounced impact, with chickens slaughtered in the hot-dry season attaining the highest weights while those in the rainy season had the lowest. Seasonal changes also affected pH, meat colour, cooking loss, and drip loss, with summer chickens displaying higher pH and lightness (L*) values, whereas the cool-dry season resulted in denser muscle fibres and firmer breast meat. Breed differences were apparent, with the Leghorn breed exhibiting the highest live weight and Austrolorp the lowest. The Orpington breed produced firmer breast meat, while Austrolorp yielded the most tender breast meat. Differences in meat colour among breeds were attributed to variations in myoglobin content and muscle activity. The findings highlight the influence of genetic, environmental, and physiological factors on village chicken meat quality, suggesting further research into nutrition and muscle fibre composition is necessary to enhance meat quality.

## Data Availability

Upon request, the corresponding author (Busisiwe Gunya) will provide the data supporting the study’s findings.
